# *Aeginetia indica* Decoction Inhibits Hepatitis C Virus Life Cycle

**DOI:** 10.3390/ijms19010208

**Published:** 2018-01-09

**Authors:** Cheng-Wei Lin, Chieh-Wen Lo, Chia-Ni Tsai, Ting-Chun Pan, Pin-Yin Chen, Ming-Jiun Yu

**Affiliations:** Institute of Biochemistry and Molecular Biology, National Taiwan University College of Medicine, Taipei 10051, Taiwan; cwlin717@gmail.com (C.-W.L.); rogerwen80@gmail.com (C.-W.L.); aaw40121@yahoo.com.tw (C.-N.T.); r05442003@ntu.edu.tw (T.-C.P.); rc810912@gmail.com (P.-Y.C.)

**Keywords:** herbal decoction, bioactive phytochemical, nutraceutical, alternative medicine, *Aeginetia indica*, hepatitis C virus (HCV), serine 235, non-structural protein 5A (NS5A), phosphorylation

## Abstract

Chronic hepatitis C virus (HCV) infection is still a global epidemic despite the introduction of several highly effective direct-acting antivirals that are tagged with sky-high prices. The present study aimed to identify an herbal decoction that ameliorates HCV infection. Among six herbal decoctions tested, the *Aeginetia indica* decoction had the most profound effect on the HCV reporter activity in infected Huh7.5.1 liver cells in a dose- and time-dependent manner. The *Aeginetia indica* decoction exerted multiple inhibitory effects on the HCV life cycle. Pretreatment of the cells with the *Aeginetia indica* decoction prior to HCV infection reduced the HCV RNA and non-structural protein 3 (NS3) protein levels in the infected cells. The *Aeginetia indica* decoction reduced HCV internal ribosome entry site-mediated protein translation activity. It also reduced the HCV RNA level in the infected cells in association with reduced NS5A phosphorylation at serine 235, a predominant phosphorylation event indispensable to HCV replication. Thus, the *Aeginetia indica* decoction inhibits HCV infection, translation, and replication. Mechanistically, the *Aeginetia indica* decoction probably reduced HCV replication via reducing NS5A phosphorylation at serine 235.

## 1. Introduction

The hepatitis C virus (HCV) is an enveloped virus of the Hepacivirus genus in the *Flaviviridae* family [1,2]. It infects approximately 170 million individuals worldwide and is a leading cause of chronic liver diseases and related complications including liver fibrosis, decompensated cirrhosis, hepatocellular carcinoma, and death [1,3].

The HCV genome is a 9.6 kb single-stranded positive-sense RNA, encoding a long open reading frame consisting of 10 proteins. These proteins are processed by host and viral proteases into individual functional proteins. Core, E1, and E2 are structural proteins that make up the viral particle. P7, NS2, NS3, NS4A, NS4B, NS5A, and NS5B are non-structural proteins required for a complete HCV life cycle. Several non-structural proteins have enzymatic functions. For example, NS3/4A is a protease involved in the processing of NS3-NS5B polyprotein into individual proteins and hence a logical target of a number of approved direct-acting antivirals (DAAs) [4]. NS5B is an RNA-dependent RNA polymerase required for viral replication and hence another logical target of DAAs [1,2]. NS5A does not have apparent enzymatic activity; however, it interacts with various cellular and viral proteins and participates in many stages of the HCV life cycle [5]. Thus, targeting NS5A is anticipated with amplified anti-HCV effects [6]. In fact, the HARVONI^®^ regimen (Gilead Sciences Inc., Foster City, CA, USA) that combines DAAs for NS5A (Ledipasvir) and NS5B (Sofosbuvir) produces a greater than 90% anti-HCV efficiency [7]. However, despite these highly efficient DAAs, eradication of HCV infection is still challenging for a number of reasons [3]. (1) The infection is often asymptomatic and hence is not identified and not treated. (2) Current treatment options are complex and are still evolving. (3) The accessibility to care and the affordability of the DAAs remain an obstacle.

NS5A consists of three domains (I, II, and III) linked by two low complexity sequence regions (LCS I and II) [5]. It is a phosphoprotein with hypo- and hyper-phosphorylated statuses. NS5A hyper-phosphorylation has been implicated in viral replication and assembly [2]. Others and we have identified several serine residues (i.e., S225, S229, S232, and S235) in the LCS I region that are responsible for NS5A hyper-phosphorylation [8,9,10,11]. Of these, S235 phosphorylation is a critical event that is indispensable for virus replication [10,11,12]. Inhibiting casein kinase I α (CKIα) responsible for NS5A S235 phosphorylation reduced S235 phosphorylation and HCV RNA levels [10]. Thus, targeting NS5A S235 phosphorylation is expected to have anti-HCV effects [12,13].

Plants are rich sources for a large number of bioactive phytochemicals with anti-HCV effects [14,15]. Here we screened six herbal decoctions for their ability to reduce HCV infection. These herbs were selected because they are produced by a number of biotech companies in Taiwan. This will ease scale-up production when desirable effects are discovered. Briefly, we describe functions/uses of each herbal decoction below. *Aeginetia indica* is used as a historical folk remedy for chronic liver diseases [16]. It also reduces renal cancer cell growth and metastasis [17]. *Angelica sinensis* contains antioxidant activities [18]. It was found to have inhibitory effects on hepatoma cells [19] and it also protects strenuous exercise-induced anemia in rats [20]. *Lycium barbarum* was found to inhibit liver inflammation and fibrosis in rats [21] and it attenuates high-fat diet-induced hepatic steatosis [22]. *Paeonia suffruticosa* was shown to protect hepatic cells by up-regulating heme oxygenase-1 [23]. *Radix curcumae* is often used to treat jaundice and cholelithiasis [24]. Dried roots of indigo plants including *Isatis tinctoria* are frequently used as an anti-inflammatory and an anti-viral agent for hepatitis, influenza, and various types of inflammation [25]. Among these six herbal decoctions tested, we found that the *Aeginetia indica* decoction exerted multiple inhibitory effects on the HCV life cycle in general and viral replication in particular.

## 2. Results

### 2.1. Aeginetia indica Decoction Inhibited HCV Reporter Activity

**Figure 1:**
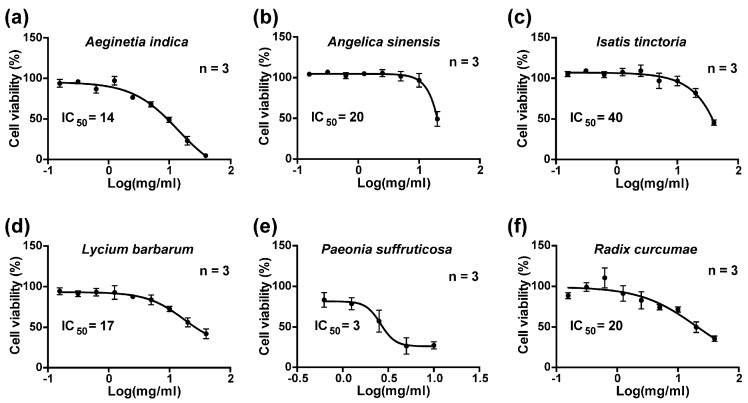
MTT-based cell viability assay for six herbal decoctions. The hepatitis C virus (HCV) host cells Huh7.5.1 were exposed to the decoction of (**a**) *Aeginetia indica*; (**b**) *Angelica sinensis*; (**c**) *Isatis tinctoria*; (**d**) *Lycium barbarum*; (**e**) *Paeonia suffruticosa*; or (**f**) *Radix curcumae* for 72 h before being assayed for cell viability. Values are mean ± SEM (standard error of the mean) summarized from three independent experiments. Estimated 50% inhibitory concentrations (IC_50_) in mg/mL are shown.

**Figure 2:**
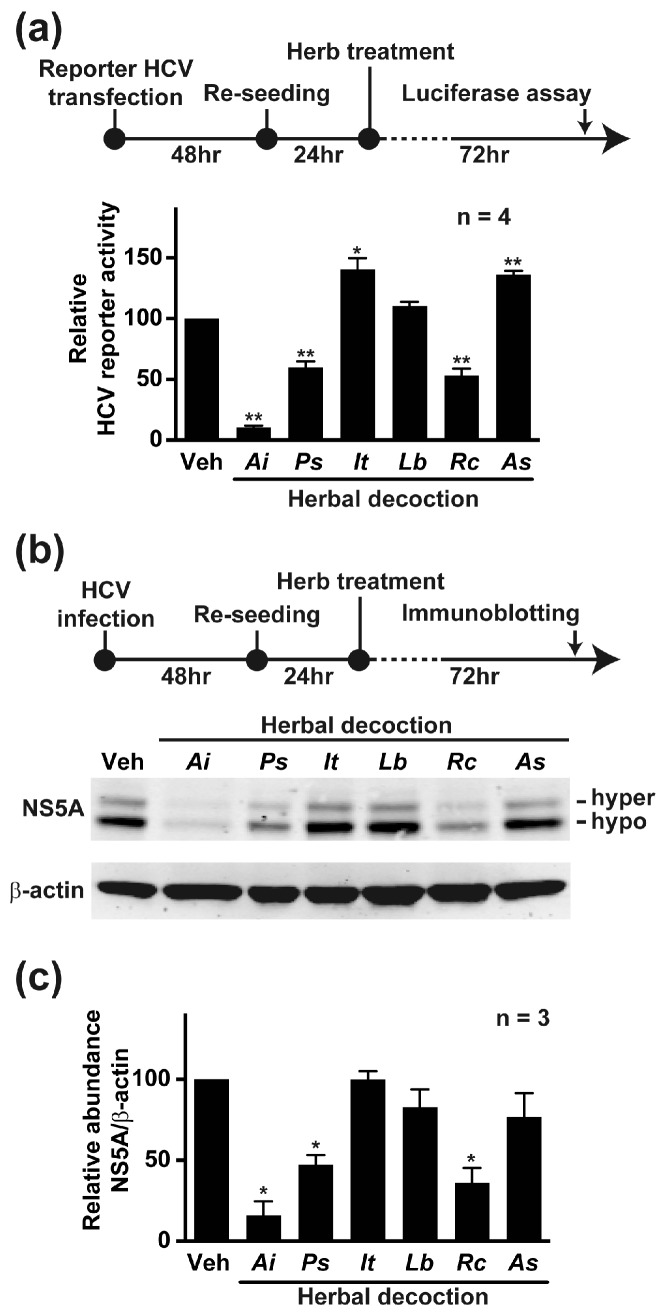
*Aeginetia indica* decoction reduced the HCV reporter activity and the HCV non-structural protein 5A (NS5A) protein level. (**a**) Effects of six herbal decoctions on the HCV reporter activity in Huh7.5.1 cells transfected with a full-length HCV genomic RNA containing a luciferase reporter (5′C19Rluc2aUbi, strain 2a). Concentrations of the herbal decoctions were (in mg/mL): *Aeginetia indica* (Ai) 8, *Paeonia suffruticosa* (Ps) 1, *Isatis tinctoria* (It) 10, *Lycium barbarum* (Lb) 10, *Radix curcumae* (Rc) 10, and *Angelica sinensis* (As) 10. Values are mean ± SEM summarized from four independent experiments. One and two asterisks indicate statistical significance at *p* < 0.05 and *p* < 0.01 based on t-test against values under the vehicle (Veh) control; (**b**) Representative and (**c**) summary of immunoblotting for HCV NS5A protein in Huh7.5.1 cells infected with HCV (J6/JFH1, genotype 2a). Hyper and hypo represent hyper- and hypo-phosphorylated NS5A, respectively.

To identify herbs with anti-HCV effects, we tested six herbal decoctions widely used in Asia for beneficial effects on the liver. Figure 1 summarizes viability of the HCV host liver cells (Huh7.5.1) exposed to the herbal decoctions for 72 h. The IC_50_ (inhibitory concentration, 50%) for *Aeginetia indica, Angelica sinensis, Isatis tinctoria, Lycium barbarum, Paeonia suffruticosa*, and *Radix curcumae* decoctions were 14, 20, 40, 17, 3, and 20 mg/mL (dry herb weight in water), respectively. About a half of the IC_50_ dose was used to test their effects on HCV reporter activity in Huh7.5.1 cells following the protocol shown in Figure 2a. Among the six herbal decoctions tested, *Aeginetia indica* reduced the HCV reporter activity by 90% (Ai) while *Paeonia suffruticosa* (Ps) and *Radix curcumae* (Rc) reduced the HCV reporter activity by 40% and 47%, respectively. *Lycium barbarum* (Lb) did not have an effect whereas *Isatis tinctoria* (It) and *Angelica sinensis* (As) elevated the HCV reporter activity by 41% and 36%, respectively. Thus, the aqueous *Aeginetia indica* decoction had the most potent anti-HCV effect among the six herbal decoctions. The reduction in the HCV reporter activity was accompanied by a paralleled reduction in the NS5A protein level as shown in the immunoblotting results (Figure 2b,c).

### 2.2. Aeginetia indica Decoction Dose- and Time-Dependently Inhibited HCV Reporter Activity

**Figure 3:**
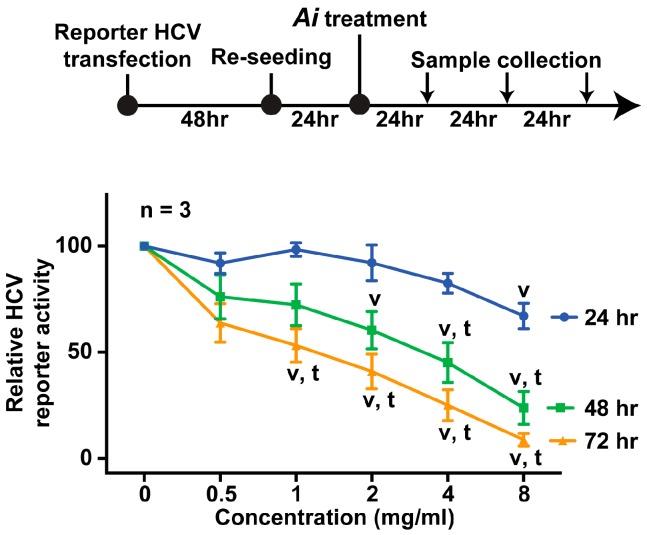
Effects of the *Aeginetia indica* decoction on the HCV reporter activity in Huh7.5.1 cells. Values are mean ± SEM summarized from three independent experiments and normalized against those under the vehicle control (0 mg/mL). Blue, green, and yellow lines represent respectively the HCV reporter activity measured at 24, 48, and 72 h after exposure to the idicated doses. Ai, *Aeginetia indica* decoction; *v*, *p* < 0.05 *t*-test against values under the vehicle control; *t*, *p* < 0.05 *t*-test against values at 24 h.

Dose- and time-dependent experiments were performed to examine the specificity of the anti-HCV effects of the *Aeginetia indica* decoction following the experimental protocol shown in Figure 3. Huh7.5.1 cells were transfected with a full-length HCV reporter RNA. Forty-eight hours later, the cells were reseeded to even out transfection variations before the cells were exposed to various doses of the *Aeginetia indica* decoction. The reporter activity was measured every 24 h up to 72 h. As summarized in Figure 3, the *Aeginetia indica* decoction dose-dependently reduced the HCV reporter activity measured at 24 (blue line), 48 (green line), or 72 (yellow line) h after exposure to the *Aeginetia indica* decoction. For any given *Aeginetia indica* decoction dose, the HCV reporter activity for the 72-h line was always the lowest, the 48-h line the middle, and the 24-h line the highest, indicating a time-dependent inhibition of the HCV reporter activity by the *Aeginetia indica* decoction.

### 2.3. Pretreating the Cells with Aeginetia indica Decoction Reduced HCV Infection

**Figure 4:**
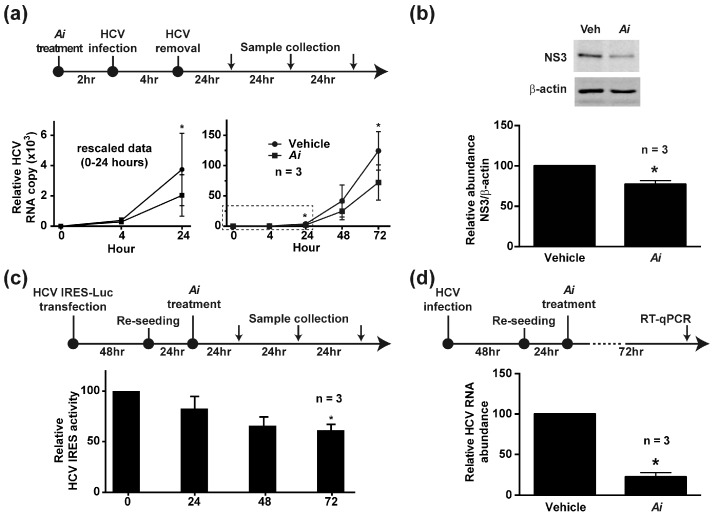
Multiple effects of *Aeginetia indica* decoction on the HCV life cycle. (**a**) HCV RNA levels in Huh7.5.1 cells pretreated with vehicle or *Aeginetia indica* decoction (8 mg/mL) followed by HCV infection. The left panel rescales datum points in the boxed area of the right panel. Values are mean ± SEM summarized from three independent experiments and normalized against the values under the vehicle control (0 mg/mL). Asterisk indicates *p* < 0.05, *t*-test against the values under the vehicle control; (**b**) Representative image and summary of immunoblotting for HCV non-structural protein 3 (NS3) protein in Huh7.5.1 cells treated the same way as in (**a**). Samples from 72 h post HCV infection were analyzed. β-actin serves as a loading control; (**c**) Effects of *Aeginetia indica* decoction on HCV internal ribosome entry site (IRES)-mediated protein translation activity in Huh7.5.1 cells; (**d**) Effects of *Aeginetia indica* decoction on HCV RNA levels in HCV-infected Huh7.5.1 cells. Values were adjusted for loading (18S RNA) and standardized against the values under the vehicle control.

To examine whether the *Aeginetia indica* decoction affects HCV virus infection, Huh7.5.1 cells were pretreated with the *Aeginetia indica* decoction for 2 h before the cells were infected with HCV for 4 h (Figure 4a). Thereafter, the cells were free of virus and free of *Aeginetia indica* decoction before the HCV RNA levels were measured with time. At 4 h post HCV infection (Figure 4a, bottom left panel), the HCV RNA copy numbers in the *Aeginetia indica* decoction-pretreated cells and vehicle-pretreated were respectively 284 and 386, indicating fewer HCV in the *Aeginetia indica* decoction-pretreated cells. These HCV RNAs started to increase as viral replication began. At 24 and 72 h post HCV infection, the HCV RNA copy numbers in the *Aeginetia indica* decoction-pretreated cells remained significantly lower than those in the vehicle-pretreated cells, consistent with an inhibitory effect of the *Aeginetia indica* decoction on HCV infection. In line with this, the amount of NS3 protein was significantly lower in the *Aeginetia indica* decoction-pretreated cells at 72 h post HCV infection (Figure 4b).

### 2.4. Aeginetia indica Decoction Inhibited HCV IRES-Mediated Protein Translation

To test whether the *Aeginetia indica* decoction affects HCV IRES (internal ribosome entry site)-mediated protein translation activity, Huh7.5.1 cells were transfected with a luciferase reporter gene driven by the HCV IRES before being exposed to the *Aeginetia indica* decoction. Figure 4c shows the protocol and the luciferase activity measurements. Compared to the vehicle, the *Aeginetia indica* decoction reduced the luciferase activity in the transfected cells, indicating an inhibitory effect of the *Aeginetia indica* decoction on the HCV IRES-mediated translation activity.

### 2.5. Aeginetia indica Decoction Reduced HCV RNA in the Infected Cells

To test whether the *Aeginetia indica* decoction affects HCV replication, Huh7.5.1 cells were infected with HCV followed by exposure to the *Aeginetia indica* decoction before the HCV RNA was measured with RT-qPCR (Figure 4d). As seen in the bar diagram, the *Aeginetia indica* decoction significantly reduced the HCV RNA levels in the infected cells, consistent with an inhibitory effect of the *Aeginetia indica* decoction on HCV replication.

### 2.6. Aeginetia indica Decoction Reduced NS5A and NS5A Phosphorylation at Serine 235

**Figure 5:**
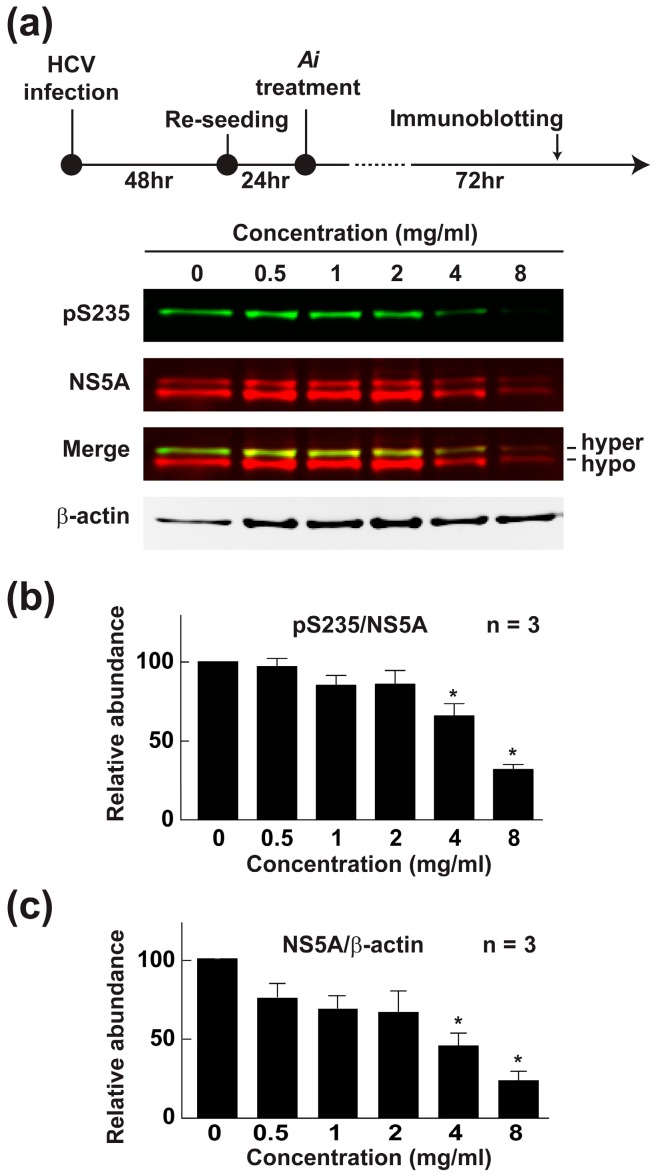
*Aeginetia indica* decoction reduced HCV NS5A phosphorylation at serine 235. Representative (**a**) and summary of immunoblotting for total NS5A (**b**) and S235 phosphorylated NS5A (**c**) in HCV-infected Huh7.5.1 cells treated with vehicle or *Aeginetia indica* decoction. Values are mean ± SEM summarized from three independent experiments and normalized against values under the vehicle control (0 mg/mL). β-actin serves as a loading control. Asterisk indicates *p* < 0.05, *t*-test against the values under the vehicle control. Ai, *Aeginetia indica* decoction; hyper, hyper-phosphorylation; hypo, hypo-phosphorylation.

Phosphorylation at serine 235 (S235) of the HCV NS5A protein is an indispensable post-translational event required for HCV replication [10,11,12]. To test whether the *Aeginetia indica* decoction reduces NS5A S235 phosphorylation, Huh7.5.1 cells were infected with HCV followed by exposure to the *Aeginetia indica* decoction and immunoblotting for NS5A protein and S235 phosphorylation (Figure 5a). As seen in the representative immunoblots, the *Aeginetia indica* decoction dose-dependently reduced NS5A phosphorylation at S235. On average, the *Aeginetia indica* decoction at 4 and 8 mg/mL significantly reduced the ratio of S235 phosphorylated NS5A over total NS5A (Figure 5b), which is consistent with an inhibitory effect of the *Aeginetia indica* decoction on NS5A S235 phosphorylation. Note that the *Aeginetia indica* decoction also reduced total NS5A (Figure 5c).

## 3. Discussion

In the present study, we found that the *Aeginetia indica* decoction had the highest anti-HCV potency among the six herbal decoctions tested (Figure 2a). The six herbs were selected because they are produced by several biotech companies in Taiwan and are used by the people for beneficial effects on the liver. In the case of HCV infection, we found that the *Aeginetia indica* decoction significantly reduced HCV reporter activity by 90% (Figure 2a). To lesser extents, the *Paeonia suffruticosa* and *Radix curcumae* decoctions also reduced HCV reporter activity. On the contrary, the *Lycium barbarum* decoction did not affect HCV reporter activity. The *Isatis tinctoria* and *Angelica sinensis* decoctions elevated HCV reporter activity. Thus, given the historical and common practice of using the above herbal decoctions, studies must be done to understand specifically under what conditions and by which mechanisms these herbal decoctions work. There are other herbal decoctions reported with anti-HCV potency [26,27]. For example, the milk thistle *Silybum marianum* decoction is frequently used by HCV patients, although with mild efficacy [28].

*Aeginetia indica* is a parasitic plant found in low elevation regions in East and Southeast Asia [29]. Previously, extracts of *Aeginetia indica* seeds was shown to induce antitumor immunity [30] and its aqueous decoction was shown to inhibit renal cancer growth and metastasis [17]. To the above effects, we added multiple inhibitory effects of the *Aeginetia indica* decoction on HCV infection (Figure 4a,b), IRES-mediated translation (Figure 4c), and RNA replication (Figure 4d). The *Aeginetia indica* decoction probably reduces viral replication by reducing NS5A phosphorylation at S235 (Figure 5b), a key phosphorylation event required for genotype 2a HCV replication [10,11]. Since S235 is a direct substrate of casein kinase I α [10,11], the *Aeginetia indica* decoction most likely inhibits casein kinase I α, thereby reducing S235 phosphorylation. Of course, the above statement requires additional experiments (e.g., in vitro kinase assays) to be 100% certain. In addition, the *Aeginetia indica* decoction also reduced the amount of total NS5A (Figure 5c). Since NS5A is a critical component of the HCV replication protein complex, the reduction of NS5A itself is expected to reduce HCV replication and assembly. Thus, reduction in total NS5A and/or NS5A S235 phosphorylation could be the mechanisms of actions of the *Aeginetia indica* decoction. Another potential mechanism is inactivation of the NF-κB-cyclooxygenase 2 axis by the *Aeginetia indica* decoction [17]. Inhibition of cyclooxygenase 2 has been reported to mediate the anti-HCV effects of *Acacia confusa* decoction and acetylsalicylic acid [31,32]. Whether the *Aeginetia indica* decoction has effects on other HCV genotypes or other viruses (Dengue and Japanese encephalitis viruses) of the *Flaviviridae* family requires further studies.

Based on the hydrophilic nature of the *Aeginetia indica* decoction, we speculated that flavonoids might be the active anti-HCV components in the *Aeginetia indica* decoction. Major flavonoids of *Aeginetia indica* have been analyzed, although not examined in the context of HCV infection [29]. Synthetic flavonoid apigenin inhibits HCV replication by decreasing the levels of mature microRNA122 required for HCV RNA stability and propagation [33,34]. Apigenin and luteolin of *Eclipta alba* exhibited dose-dependent inhibition of HCV replicase in vitro and anti-HCV replication activity in the cell culture system [35]. Moreover, anthocyanin cyanidin 3-*O*-rutinoside of black rice could potentially inhibit HCV viral assembly by reducing hepatic lipogenesis [36], a process that facilitates association of the HCV core protein with the lipid droplets where viral assembly takes place [37]. We attempted to isolate the active anti-HCV components from the *Aeginetia indica* decoction; however, fractionation of the decoction significantly reduced the efficacy. This is a rather common phenomenon for herbal decoctions, which often contain multiple bioactive phytochemicals and thereby exert multiple effects. Separation of the active phytochemicals often reduces the overall efficacy.

## 4. Materials and Methods

### 4.1. Cells and HCV Constructs

**Figure 6:**
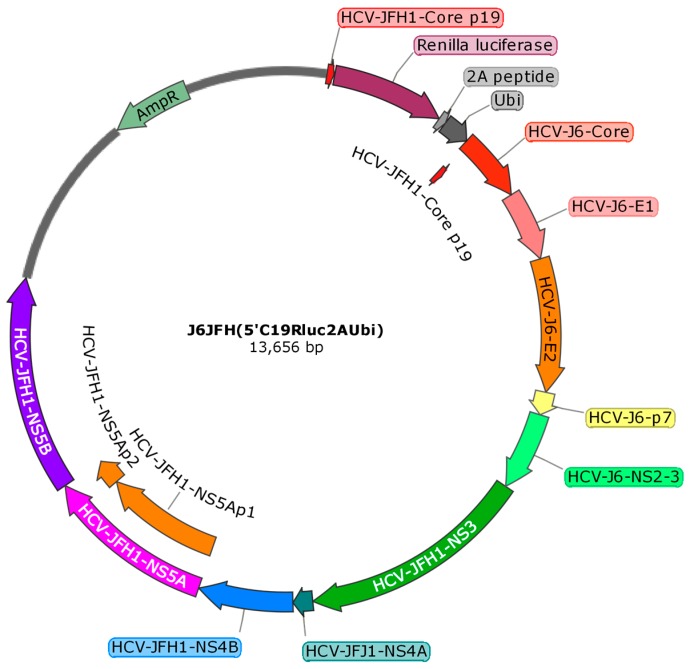
Scheme of the HCV reporter construct J6JFH (5′C19Rluc2AUbi).

Human hepatocarcinoma 7.5.1 cell line (Huh7.5.1) was maintained in Dulbecco’s modified minimal essential medium (DMEM) supplemented with 10% fetal bovine serum without any antibiotics in a 5% CO_2_ incubator at 37 °C. The HCV Renilla luciferase reporter (J6/JFH1) construct (5′C19Rluc2aUbi) was from Charles Rice at the Rockefeller University (New York, NY, USA) [38]. It is a monocistronic reporter virus DNA construct composed of DNA of the J6 and JFH1 isolates. It has the JFH1 5′ UTR followed by amino acids 1–19 of the J6 core protein (Core p19), Renilla luciferase, foot and mouth disease virus autoproteolytic 2A peptide, a ubiquitin monomer (Ubi), and the entire HCV polyprotein coding sequence starting with the first amino acid (Met) of the core protein (Figure 6). The HCV polyprotein coding sequence has an intragenotypic break point between the non-structure proteins NS2 of the J6 isolate and NS3 of the JFH1 isolate. The construct also carries β-lactamase gene for ampicillin resistance (AmpR). The IRESHCV-LUC construct was from Penelope Mavromara at the Hellenic Pasteur Institute, Greece [39].

### 4.2. Herbal Decoction

The herbs were approved and decocted by Sun Ten Pharmaceutical Company (Taipei, Taiwan). In brief, dry plant materials were finely ground and the decoctions were prepared by boiling 250 g of the plant materials in 1250 mL of water for 30 min. The decoctions were concentrated to 250 mL with an evaporator at room temperature. The decoctions were centrifuged at 3000 rpm for 10 min, filtered through a 0.45 µm syringe filter, stocked at a concentration of 1 g/mL, and stored at −20 °C until use.

### 4.3. Cell Viability Assay

The Huh7.5.1 cells were seeded in a 96-well plate at a density of 14,000 cells/well one day before being exposed to the herbal decoctions for 3 days. The medium was changed daily with fresh herbal decoction-containing medium. To assess cell viability, the medium was removed and 50 µL MTT reagent (3-(4,5-dimethylthiazol-2-yl)-2,5-diphenyltetrazolium bromide, catalog No. M2003, Merck Ltd., Taipei, Taiwan) was added to each well. After 40 min of incubation in 5% CO_2_ and at 37 °C, 100 µL dimethyl sulfoxide (DMSO) (catalog No. D8418, Merck Ltd., Taipei, Taiwan) was added to each well. After rocking for 15 min on an orbital shaker, the 96-well plate was subjected to 540 nm absorbance measurements.

### 4.4. HCV RNA Preparation

The HCV construct was linearized via Xbal (catalog No. R105, New England Biolabs, Ipswich, MA, USA) digestion and used to transcribe the viral RNA using the Ambion MAXIscript T7 in vitro transcription kit (catalog No. AM1312, Ambion, Waltham, MA, USA). The viral RNA was purified by phenol-chloroform extraction and isopropanol precipitation. Quality and quantity of the RNA was assessed by a NanoDrop spectrophotometer (Thermo Fisher Scientific Inc., Waltham, MA, USA).

### 4.5. HCV Reporter Activity Assay

Huh7.5.1 cells were seeded at a density of 2 × 10^6^ cells in a 10-cm petri dish one day before being transfected with 10 µg HCV RNA with the DMRIE-C Reagent (catalog No. 10495-014, Thermo Fisher Scientific Inc., Waltham, MA, USA). Forty-eight hours after the transfection, the cells were freed from the petri dish with trypsin digestion and reseeded into a 6-well plate (4 × 10^5^ cell/well) or a 48-well plate (4 × 10^4^ cell/well). The cells were then exposed to vehicle or herbal decoctions for 3 days before they were lysed and subjected to the luciferase activity assay using a luciferase assay kit (catalog No. E1500, Promega, Madison, WI, USA). For dose dependence and time course studies, the cells were treated with vehicle or herbal decoctions at various concentrations and time before the cells were lysed for the luciferase assay.

### 4.6. Infectious HCV Virion Production

In vitro-transcribed HCV RNA was transfected into the Huh7.5.1 cells for 3 days before the virion-containing culture medium was collected daily for a week. The medium was pooled and centrifuged at 3000× *g* for 30 min to remove cell debris before the virion in the supernatant was concentrated with a 100 kDa Amicon Ultra-15 centrifugal filter (UFC910008, Merck Ltd., Taipei, Taiwan). Viral titer was determined with the method of serial dilution (10×) that resulted in infection (i.e., NS5A positive) and detected with immunofluorescence staining using the 2F6 antibody (BioFront Technologies, Tallahassee, FL, USA). For virus infection, Huh7.5.1 cells were infected with the virion with a multiplicity of infection at 0.001. Specific conditions are illustrated in the figures.

### 4.7. Immunoblotting

Huh7.5.1 cells were lysed in the IP lysis buffer (8 M Urea, 75 mM NaCl and 50 mM Tris, pH 8.0). Protein concentration was measured with a BCA assay kit (catalog No. TAAR-ZBE6, TOOLS, New Taipei City, Taiwan). In general, 20 µg of protein was separated on a 7.5% SDS-PAGE gel and transferred to a nitrocellulose membrane (catalog No. 162-0112, BOR-RAD, Hercules, CA, USA). The membrane was blocked in a blocking buffer (phosphate-buffered saline containing 1% bovine serum albumin) before the proteins of interest were detected with anti-actin (A5441, Merck Ltd., Taipei, Taiwan), anti-NS3 (2E3, BioFront Technologies, Tallahassee, FL, USA), anti-NS5A (7b5, BioFront Technologies, Tallahassee, FL, USA), or anti-S235 phosphorylation primary antibodies [10], followed by infrared conjugated secondary antibodies (IRDye 680 and IRDye 800) obtained from LI-COR (Lincoln, NE, USA). Protein intensity was quantified with the ODYSSEY fluorescence imaging system and software (Version 3.0.21, LI-COR, Lincoln, NE, USA).

### 4.8. HCV IRES-Mediated Translation Activity Assay

Huh7.5.1 cells were transfected with the IRESHCV-LUC construct. Two days later, the cells were freed from the petri dish with trypsin digestion and reseeded into a 6-well plate at a density of 3.5 × 10^5^ cells per well. One day later, the cells were treated with vehicle or herbal decoctions for 3 days before the luciferase activity assay.

### 4.9. HCV RNA Measurement

Total RNA from the Huh7.5.1 cells were extracted with the Direct-zol™ RNA MiniPrep (catalog No. R2052, ZYMO RESEARCH, Irvine, CA, USA) and quantified with RT-qPCR. First strand cDNA was synthesized with the SuperScript III Reverse Transcriptase (catalog No. 18080-044, Thermo Fisher Scientific Inc., Waltham, MA, USA) plus random hexamer primer (catalog No. N8080127, Thermo Fisher Scientific Inc., Waltham, MA, USA) or HCV gene-specific primer (5′-CACTCGCAAGCACCCTATCA-3′). The KAPA SYBR FAST qPCR master mix (catalog No. KK4604, KAPA BIOSYSTEMS, Wilmington, MA, USA) was used to quantify the RNA abundance with HCV-specific 5′ UTR primers (sense, 5′-TCTGCGGAACCGGTGAGTA-3′; antisense, 5′-TCAGGCAGTACCACAAGGC-3′) or 18S rRNA primers (sense, 5′-AAACGGCTACCACATCCAAG-3′; antisense, 5′CCTCCAATGGATCCTCGTTA-3′).

## 5. Conclusions

Among six commonly used herbal decoctions thought to benefit the liver, we found that the *Aeginetia indica* decoction contains factors that exert multiple inhibitory effects on the hepatitis C virus life cycle. The most profound effect of the *Aeginetia indica* decoction was inhibition of hepatitis C virus replication likely via reducing phosphorylation of the non-structural protein 5A at serine 235.
